# Notes on the Pneumogastric

**Published:** 1873-11

**Authors:** J. F. Alleyne Adams

**Affiliations:** Pittsfield


### Selections.

*Notes on the Pneumogastric.* By J. F. ALLEYNE ADAMS, M.D., Pittsfield. Read before the Massachusetts Medical Society, June 3, 1873.

[Continued from page 627.]

*Influence of the Pneumogastric upon the Stomach.*—As early as 1801, Bichat observed that irritation of the pneumogastrics produced contraction of the muscular coat of the stomach. This fact has been confirmed by Tiedemann, Gmelin, and more recently by Longet. The last named author has likewise observed that these contractions are very marked during stomach digestion, but are wanting when the stomach is empty. He has also found that irritation of the splanchnic nerves, while it produces movements in the intestines, does not affect the stomach. When the pneumogastrics are irritated, the contractions of the stomach are tardy, resembling the action of the sympathetic nerves upon unstriped muscular tissue. From these facts, Longet concludes that the motor nerve-filaments of the stomach, although contained in the pneumogastrics, are derived by anastomosis from the sympathetic system. We have already seen that the pneumogastrics receive, in the neck, a large supply of sympathetic fibres, from the cervical ganglia.

The effects of *section* of the pneumogastrics upon the stomach have been clearly demonstrated by Bernard. He found that upon division of these nerves, the contractions of the muscular walls instantly ceased, the mucous membrane lost its turgescence and became pale, the secretion of gastric juice was arrested, and the sensibility of the organ was abolished. Schiff, however, has found that the movements of the stomach, although immensely diminished in activity, are not entirely abolished, for substances may still be very slowly passed to the pylorus. These movements he attributes to local irritation of the intramuscular terminal nervous filaments. It has been asserted by Tiedemann and Gmelin, that digestion, arrested by section of the pneumogastrics, may be, to a certain extent, re-established by galvanization of the peripheral extremities of the divided nerves.

Section of the pneumogastrics effectually prevents the action of emetics and cathartics. This fact has been established, and its causes most thoroughly investigated, by Prof. Horatio C. Wood, Jr., of Philadelphia, in a most elaborate and conclusive series of experiments.* These were fifty-one in number, upon dogs, cats

* Horatio C. Wood, Jr., "On the Influence of Section of the Cervical Pneumogastrics upon the Action of Emetics and Cathartics." *American Jour. of Medical Sciences.* Vol. ix. 1870.

and rabbits, most of them being vivisections; and the results may be concisely stated as follows:

1. After section of the pneumogastrics, the largest doses of such emetics and cathartics as arsenic, tartar emetic, veratria, croton oil, gamboge and calomel, with rare exceptions, failed to produce vomiting or purging.

2. In these cases, there was no secretion of gastric or intestinal mucus, whose production in large quantities is the usual result of these drugs, and is essential to their action.

3. These results are not due to non-absorption of the medicines, for these and other poisons produce death quite as speedily as when the nerves are not divided.

4. The absence of purging cannot be explained by failure of muscular action, for the peristaltic action of the bowels continues after section of the pneumogastrics.

5. One probable explanation of this phenomenon lies in the accumulation of carbonic acid in the blood; for animals, hypodermically injected with veratria, but with nerves intact, immersed in an atmosphere of carbonic acid, died without vomiting or purging. This cannot be the sole cause, however, for enough carbonization of the blood is required to produce insensibility, while, after section of the nerves, vomiting and purging are prevented, long before this stage is reached.

6. A second cause is probably the interference with the circulation of the lungs backing up the blood in the pulmonary artery, consequently in the right heart, and finally in the portal circulation, arresting the action of the mucous glands.

7. A third cause is perhaps shock.

8. The opposite results obtained by Brodie and Reed may be explained by the occasional failure of section of these nerves to affect the pulmonary circulation.

Dr. Wood, therefore, attributes the stoppage of vomiting and purging by section of the pneumogastrics entirely to arrest of the secretory function of that nerve, but seems to leave out of the question the reflex function of the medulla oblongata, by which gastric irritation is transmitted from the pneumogastric to the phrenic nerve, and which doubtless contributes to emesis, in those cases where the emetic is given by the stomach. In these cases, the irritation of the emetic, transmitted to the medulla by the centripetal fibres of the vagus, excites simultaneously the secretory filaments of that nerve, filling the stomach with mucus, and the excito-motor fibres of the phrenic, causing spasmodic contraction of the diaphragm. In cases of cerebral vomiting, the emetic influence seems to be transmitted, at first, entirely through the phrenic, hypersecretion not commencing until the stomach is emptied of its alimentary contents; and, in the case of some of the most prompt emetics, such as sulphate of zinc and turpeth mineral, the reflex action upon the phrenic doubtless precedes that upon the secretory filaments of the pneumogastric.

In this connection, it will be interesting to consider the relation of the pneumogastric to the etiology of migraine or sick-headache, especially since the subject has been so clearly and beautifully brought to the notice of the profession by Dr. Anstie in his work on neuralgia, and also in recent numbers of the *Practitioner*.* Concerning the nature of this distressing and obstinate affection, the most diverse opinions have been and are still held. Of recent writers, we find Du Bois Reymond maintaining that it is due to a tetanic condition of the vaso-motor nerves of the affected side of the head, the pain being analogous to that felt during tetanic contraction of voluntary muscles, or the unstriped muscles of the uterus, intestines, etc. Moellendorf, on the other hand, thinks that hemicrania is based on the appearance of a paralytic condition of the vaso-motor nerves controlling the carotid artery of one side, which causes relaxation and distension of the arteries of the brain. Dr. Anstie differs from both these writers in calling migraine a trigeminal neuralgia, a view which has, of late years, been pretty generally held by the profession in England. There is one essential point, however, in which Dr. Anstie takes an independent position, and that is, in locating the primary lesion in the medulla oblongata, instead of the chylo-poietic viscera, as is more commonly believed. Migrainous pain, he contends, means an atrophic molecular irritation in the trigeminus root, and migrainous vomiting means a similar process in the vagus root. That is to say, an irritation in the floor of the fourth ventricle, being propagated in one direction by the trigeminus, gives rise to pain, and in another direction by the vagus, upsets the stomach. He admits the frequency of simple dyspeptic headaches, with vomiting and a foul tongue; but a true migraine he describes as a trigeminal neuralgia, complicated with vomiting, though the tongue is clean, occurring at regular intervals, induced by over-fatigue, and not by over-eating, beginning in early life in persons whose family history shows nervous disease, and passing off in middle life, or merging into some other form of nervous affection, as epilepsy, asthma, angina pectoris, or persistent facial neuralgia. In accordance with this view, his treatment is strongly tonic; fasting is discountenanced, and the patients are put upon an abundant diet, with cod-liver oil. Drastic cathartics are considered pernicious, as weakening the digestive power, and reducing the general strength. The indications during an attack, he maintains, are entirely for the relief of pain and production of sleep. For this purpose, he relies chiefly upon caffeine, given by the mouth if it can be borne, otherwise

* F. E. Anstie. Neuralgia and its Counterfeits. London: 1871.

Ibid. A Clinical Lecture on Migraine. *Practitioner*, November and December, 1872.

hypodermically. If this fail, he resorts to the hypodermic use of morphia. Dr. Allbutt* replies to Dr. Anstie, contending that gastro-hepatic disturbance plays the most important part in the production of this affection; but the difference between these two gentlemen seems to be chiefly one of classification, for the cases which Allbutt considers typical are not admitted by Anstie to be true migraine. In this country, I am sure but a small minority of the sick-headaches can be classed under Anstie's definition of migraine, for the most frequent cases appear to be such as Chambers† so graphically describes, and which are dependent upon the centripetal rather than the centrifugal function of the pneumogastric.

The researches of Dr. Waller, of Geneva,‡ upon the effects of compression of the cervical pneumogastrics, for the relief of various nervous affections, have been reserved for the close of this paper, as they bear upon all three of its divisions. Although the cases reported are few, they possess a strong interest, both in reference to the importance of the results and the physiological mechanism whereby these results are obtained. He reports five cases, illustrating the effects of pressure upon persons in health, and four cases in which it was applied as a therapeutic agent. The pressure is applied with the thumb, over the carotid artery on one side, usually a little below the angle of the jaw, the thumb being moved until a sensitive spot is found, which he considers indicative of the presence of the vagus. It will be remembered that the nerve lies in the posterior groove between the carotid artery and the internal jugular vein. The symptoms produced in his own case are thus described: "A moderate pressure soon occasions a deep-seated sensation, of a peculiar benumbing character, in the head, which scarcely amounts to pain." Then follows "a sense of languor and of fainting, as if 'going off,' which is so manifest that, if the head and body be not supported, and if pressure on the nerve be continued, complete syncope ensues. Simultaneously, other symptoms of a respiratory, cardiac and gastric nature make their appearance. The respiration, at first arrested for a few moments, becomes heaving and retarded; the heart's action, depressed in force, is disturbed and irregular, as may be felt by the pulsation of the carotid, under the finger. The gastric symptoms are marked uneasiness over the stomach, sometimes amounting to nausea and even vomiting."

In the other cases, the effects were similar, but the heart's action was reduced in frequency as well as in force. In two of them, the

* Clifford Allbutt on Migraine. *Practitioner*, January, 1873.

† On the Indigestions. 1870.

‡ Augustus Waller. On the Effects of Compression of the Vagus Nerve, in the Cure or Relief of Various Nervous Affections. *Practitioner*, April, 1870.

cheek and ear of the side upon which pressure was made were observed to be paler and of lower temperature than those of the opposite side; and in one, there was slight protrusion of the eye-ball.

These symptoms Waller attributes to an irritation by pressure of the trunk of the pneumogastric and the cervical cord of the sympathetic, which lies beneath it, so that it is impossible to make firm pressure upon the one without also pressing upon the other. By comparing these symptoms with the effects of galvanization of these nerves, we find them to be identical. The retardation of the heart's pulsations, and the slow, heaving respiration, result from irritation of the inhibitory nerve-fibres of those organs in the vagus; the diminution of arterial pressure is due to irritation of the depressor nerve, and the nausea may arise from irritation of gastric nerve-fibres, or may be wholly or in part dependent upon the faintness. The pallor and reduction of temperature of the cheek and ear, and protrusion of the eye-ball, are symptoms referable to irritation of the sympathetic, being among the characteristic symptoms of Basedow's disease, an affection whose primary lesion is in the cervical sympathetic ganglia, or the adjacent cilio-spinal centre of the spinal cord. The peculiar benumbing sensation in the head is probably caused by cerebral anæmia, from irritation of vaso-motor nerves. With regard to the faintness and nausea, it is difficult to say which nerve contributes the most to them, for they may be induced by galvanization of either the vagus or sympathetic. Waller lays the most stress upon the irritation of the vagus; but, as it is impossible to press upon this, without also pressing the sympathetic cord, the two sets of symptoms are necessarily simultaneous and liable to confusion.

Previous writers, who had made use of pressure upon the neck, supposed its effects to be due to obstruction of the arterial or venous circulation, and Dr. Waller himself shared this belief until 1861, when he became convinced that these phenomena are of purely nervous origin. Dr. Parry, of Bath, was the first to apply practically, in the early part of this century, what he termed compression of the carotids, for inducing sleep, and for the immediate relief of attacks of hysteria and epilepsy; but long before his day, Aristotle had written "that if the veins of the neck become compressed exteriorly, one sees a man shut his eyes and fall down senseless, as if he were strangled, although he is not so." But Waller shows that compression of the carotid arteries will not induce the symptoms described, adducing the fact that, in animals, both carotids may be tied, without producing any disturbance of the heart's action, or of innervation. In man, too, the ligation of one of the carotids gives rise, in the great majority of cases, to no immediate symptoms of a marked character, the secondary symptoms which occasionally supervene, such as convulsions, coma, collapse, etc., being of a totally different nature, and generally attributable to lesions of the brain. In those rare cases in which impaired eyesight and syncope have immediately followed the ligation of the artery, it is likely that the vagus or sympathetic nerves, or both, have been included in the ligature; for, when such is the case in animals, the symptoms are found to be similar. Ligation of the internal jugular vein has also been frequently performed, without any peculiar symptoms resulting, although a case is within my knowledge where ligature of one of these veins, for hemorrhage, was followed by coma of three days' duration. Even in this case, it is not unlikely that the nerves were included in the ligature. Simultaneous compression of both internal jugulars would undoubtedly produce some cerebral congestion, whose symptoms would, however, differ essentially from those described by Waller; but it is probable, from the manner in which the pressure is made by him, that the circulation in these veins is but slightly, if at all, impeded. Taking these circumstances in connection with the known effects of irritation of the vagus and sympathetic nerves, we may safely conclude that Waller's views are sound, and a valuable addition to medical science.

The cases which he reports, in which a therapeutic application was made of compression of the pneumogastric, are not sufficiently numerous for generalization, but are very striking and suggestive. In one of them, violent nervous disturbance of the heart, with hysterical symptoms, came on in a lady suffering from an attack of acute rheumatism. Compression was made, first of one vagus, and afterwards of both. In a few minutes, the agitation had subsided, the heart's action became quieter and more regular, and finally, all the nervous symptoms subsided. A few minutes afterward she sank into a steady sleep, and, on awakening, was perfectly quiet, and the cardiac symptoms never re-appeared.

Another case was that of a lady, who, in consequence of a fall on the previous day, had severe neuralgia of the head and back, hyperæsthesia of the ulnar and median nerves, much mental excitement, a small pulse, at eighty, and frequent fits of obstinate vomiting. Pressure was applied to the left vagus. The result was perfect quietude, with laborious breathing, for about ten minutes; pulse weak and irregular. After removing the pressure, the pulse fell to seventy, and became full and regular, the patient lying quiet, and apparently unconscious, for upwards of ten minutes more. When she came to her senses, the vomiting had ceased, and did not return. The pain and excitability had left. The fullness of the pulse remained for some hours.

A third case was that of a lady with an irritable stomach, the sequela of gastritis, who had frequent attacks of spasmodic vomiting. One of these attacks was quieted by Dr. Waller, by pressure on one of the vagi, and subsequently the patient acquired the habit of making the pressure herself, when nausea came on, and always with the effect of quieting the stomach and preventing emesis.

The fourth and last case was that of a young lady who had suffered for several months from a severe hemicrania, recurring periodically, every day. In this case, Dr. Waller resolved to make use of pneumogastric compression as a "perturbating" agent, as he expresses it, and, to that end, while the patient was in the sitting posture, made strong compression of both vagi. Syncope occurred immediately, and she remained quiet and unconscious for two hours, although the respiration and heart's action were regular. The result was, that she had had, in the two years intervening between this treatment and the report of the case, not the slightest return of the hemicrania.

We ought not to leave Waller's pressure method without referring to Rothroch's cases of the therapeutic application of *ice* to the neck, in the region of the pneumogastric.* This was done in cases of hysterical delirium, one being idiopathic, and the other following the use of an anæsthetic; and also in a case of suspended respiration, in etherization.

In all the cases, the application was immediately followed by a deep, heaving respiration, and restoration to consciousness, with muscular relaxation. There was no faintness, however, as is the case when pressure is made; and no mention is made of any retarding of the heart's action. It is difficult to tell in what way the ice acted, in these cases. Dr. Rothroch supposes that it, in some way, supplied the *besoin de respirer*, and that shock, also, was a not unimportant factor. Shock, however, was not the sole agency, for when the ice was applied to the *head*, no effect was produced. This mode of treatment is so easily applied, that it is to be hoped it may be given a general trial.

In closing this paper, it need only be said, that the fear of adding unduly to its length has necessitated the omission of much that properly belongs to the subject, such as the relations of the pneumogastric to the larynx and to the liver, and the various forms of pneumogastric neuralgia. The object of the essay will have been accomplished, if it has succeeded in reviewing, with clearness and accuracy, the most recent investigations concerning some of the more obscure functions of the vagus; and, while showing what brilliant results have already been accomplished, indicating the yet unopened mines of wealth that remain for the reward of future explorations.—*Boston Med. and Surg. Journal.*

* J. T. Rothroch. Ice to the Neck in Hysteria, and also in Suspended Respiration. *Philadelphia Medical Times*, Vol. iii, No. 53, p. 67.

